# Epithelial down‐regulation of the miR‐200 family in fibrostenosing Crohn's disease is associated with features of epithelial to mesenchymal transition

**DOI:** 10.1111/jcmm.13836

**Published:** 2018-09-06

**Authors:** Shameer J. Mehta, Amy Lewis, Anke Nijhuis, Rosemary Jeffery, Paolo Biancheri, Antonio Di Sabatino, Roger Feakins, Andrew Silver, James Oliver Lindsay

**Affiliations:** ^1^ Centre for Genomics and Child Health Blizard Institute Barts and The London School of Medicine & Dentistry London UK; ^2^ Centre for Immunobiology Blizard Institute Barts and The London School of Medicine & Dentistry London UK; ^3^ Norwich Medical School University of East Anglia Norwich UK; ^4^ Department of Internal Medicine San Matteo Hospital University of Pavia Pavia Italy; ^5^ Department of Histopathology The Royal London Hospital London UK

**Keywords:** Crohn's disease, epithelial to mesenchymal transition, fibrosis, miR‐200 family

## Abstract

Intestinal mesenchymal cells deposit extracellular matrix in fibrotic Crohn's disease (CD). The contribution of epithelial to mesenchymal transition (EMT) to the mesenchymal cell pool in CD fibrosis remains obscure. The miR‐200 family regulates fibrosis‐related EMT in organs other than the gut. E‐cadherin, cytokeratin‐18 and vimentin expression was assessed using immunohistochemistry on paired strictured (SCD) and non‐strictured (NSCD) ileal CD resections and correlated with fibrosis grade. MiR‐200 expression was measured in paired SCD and NSCD tissue compartments using laser capture microdissection and RT‐qPCR. Serum miR‐200 expression was also measured in healthy controls and CD patients with stricturing and non‐stricturing phenotypes. Extra‐epithelial cytokeratin‐18 staining and vimentin‐positive epithelial staining were significantly greater in SCD samples (*P* = 0.04 and *P* = 0.03, respectively). Cytokeratin‐18 staining correlated positively with subserosal fibrosis (*P* < 0.001). Four miR‐200 family members were down‐regulated in fresh SCD samples (miR‐141, *P* = 0.002; miR‐200a, *P* = 0.002; miR‐200c, *P* = 0.001; miR‐429; *P* = 0.004); miR‐200 down‐regulation in SCD tissue was localised to the epithelium (*P* = 0.001‐0.015). The miR‐200 target *ZEB1* was up‐regulated in SCD samples (*P* = 0.035). No difference in serum expression between patient groups was observed. Together, these observations suggest the presence of EMT in CD strictures and implicate the miR‐200 family as regulators. Functional studies to prove this relationship are now warranted.

## 1 INTRODUCTION

Crohn's disease (CD) is a chronic, inflammatory, relapsing and remitting condition that predominantly affects the gastrointestinal tract. Research focussed on intestinal barrier function, host immune responses, genetic susceptibility and the gut microbiota has advanced our understanding of the aetiopathogenesis of inflammatory CD. In contrast, the mechanisms responsible for the formation of fibrotic strictures in fibrostenosing CD are not well defined. The estimated lifetime risk of surgery for patients with CD is approximately 70%^1^, ^2^ and fibrostenosing disease is the most common surgical indication. Surgery is not only costly for healthcare systems^3^ but is associated with unemployment and poorer quality of life scores for patients.^4^ Current medical therapies are unable to reverse established fibrosis and the prediction of stricture development remains a significant clinical challenge. Moreover, the rates of progression to stricturing disease have not altered despite the introduction and subsequent widespread use of biological therapy.^5^

Intestinal mesenchymal cells play a pivotal role in deposition of extracellular matrix that characterises fibrosis in CD.^6^, ^7^ Manipulating the accumulation and function of intestinal mesenchymal cells is an attractive therapeutic option for fibrostenosing CD. Potential mechanisms of mesenchymal cell accumulation include expansion of the resident cell population, recruitment of bone marrow‐derived circulating fibrocytes, and epithelial to mesenchymal transition (EMT).^8^ EMT is a process in which epithelial cells lose cellular adhesion and acquire mesenchymal morphology, together with an increased migratory phenotype. Epithelial‐specific cellular markers are lost whilst mesenchymal markers are gained. The loss of e‐cadherin, a cell adhesion molecule responsible for adherens junction integrity,^9^, ^10^ appears to be a necessary step for EMT to occur.^11^

EMT has been heavily implicated in both cancer progression (type 3 EMT)^12^, ^13^, ^14^ and the development of tissue fibrosis (type 2 EMT). The latter has been demonstrated in renal,^15^, ^16^, ^17^, ^18^, ^19^ hepatic,^20^, ^21^, ^22^ pulmonary^23^, ^24^ and cardiac^25^, ^26^ models. Two recent studies of type 2 EMT have provided evidence for the concept of “partial EMT”^27^, ^28^ whereby cells lose epithelial characteristics but promote fibrosis by means other than the active deposition of extracellular matrix within the deeper tissue layers. These include the secretion of exosomes and cytokines to promote the differentiation of fibroblasts to myofibroblasts and the recruitment of bone‐marrow derived mesenchymal cells. In one study these transitioning cells were found close to the epithelial layer from which they had originated.^27^ This concept of “partial EMT” is thought occur predominantly in type 2, rather than type 3, EMT.^27^, ^29^ It is possible that this is the case in intestinal fibrosis, although comparatively few studies have been performed examining the role of EMT in this setting. Cell fate mapping in a murine colitis model demonstrated double‐labelled intestinal epithelial cells for both epithelial‐specific and mesenchymal‐specific cellular markers.^30^ In addition, high levels of nuclear β‐catenin localisation and expression of the transcription factor *SLUG,* which can both be associated with EMT, were reported in human CD intestinal samples.^31^

MiRNAs regulate EMT by negative post‐transcriptional control of target gene expression. The miR‐200 family impacts on fibrosis‐related EMT in organs other than the intestine.^32^, ^33^, ^34^, ^35^ MiR‐200b overexpression leading to suppression of TGFβ‐induced EMT alterations has been demonstrated in an intestinal epithelial cell line.^36^ In addition, down‐regulation of the miR‐200 family and altered expression of the EMT effectors *SNAIL* and *SLUG* has been reported in colonic tissue from patients with CD.^37^ In this study, we examined well‐characterised intestinal CD specimens for the presence of EMT by quantifying cellular marker expression and related this to the histological degree of fibrosis. We also confirmed dysregulation of the miR‐200 family and downstream effectors in fibrotic samples and demonstrate that the former is specific to the intestinal epithelium. Finally, we assessed serum levels of the miR‐200 family in different CD phenotypes.

## 2 METHODS

### 2.1 Patient recruitment

Patients at Barts Health NHS Trust with a documented history of stricturing ileal CD undergoing surgical resection were recruited. Any patient with fistulating disease, dysplasia or cancer (previous or current) was excluded. Patients were consented under ethical approval granted by the Health Research Authority (REC reference 13/LO/1292) specifically for this study. Data on age, gender, smoking status, disease duration, disease location, medications, previous surgery and inflammatory markers were collected. Areas of stricture (SCD) and non‐stricture (NSCD) were identified and harvested by histopathologists then stored in RNA Later (Qiagen, UK) prior to RNA extraction.

For the serum miRNA expression studies, samples from healthy controls (n = 13) and patients with CD (n = 42) were provided, along with a database of clinical characteristics, by colleagues from the Department of Internal Medicine, San Matteo Hospital (Pavia, Italy) as described previously.^38^ CD patients were either classified as SCD (with a stricturing phenotype) or NSCD (with either an inflammatory or penetrating phenotype).

### 2.2 Immunohistochemistry

Archived small intestinal resection specimens from patients with stricturing CD were identified from the Barts Health NHS Trust histopathology database. Pathology reports were interrogated and blocks containing SCD and NSCD tissue for each patient chosen. 4 μm thick sections of formalin‐fixed paraffin‐embedded (FFPE) tissue were cut followed by de‐waxing, hydration and blocking in goat serum. Primary antibodies used were anti‐e‐cadherin (Dako Clone NCH‐28) at 1:50 dilution and 30 minutes antigen retrieval in pH9 buffer; anti‐cytokeratin 18 (Dako Clone DC10) at 1:100 dilution and 20 minutes antigen retrieval in pH9 buffer; anti‐vimentin (Dako, Glostrup, Denmark) at 1:200 dilution and 30 minutes antigen retrieval in pH9 buffer. Human adult appendix tissue served as positive controls. Negative controls omitting the primary antibodies were also included.

### 2.3 Immunohistochemistry image analysis

Stained slides were scanned with the NanoZoomer 2.0 slidescanner (Hamamatsu, Japan). Tissue areas of interest for each cellular marker were outlined in ImageJ software in order to block out the rest of the image. Predetermined thresholds for pigment colour on the red‐green‐blue spectrum, size and circularity were then applied to these areas of interest in order to capture both background cellular counterstaining and positive cellular antibody staining separately (Table S1). These thresholds were used consistently across all samples. Images generated by the ImageJ program were also checked visually to ensure staining included in the analysis was truly cell‐related and not simply as a result of cellular debris or tissue artefact. Extra‐epithelial staining of e‐cadherin and cytokeratin‐18 was measured using three randomly selected fields of lamina propria and submucosa at 20× magnification of each sample and measuring the proportion of positive cellular staining from total counterstaining. Vimentin staining was measured in 2 randomly selected regions of 100 consecutive surface epithelial cells as well as 3 randomly selected crypts per slide, again at 20× magnification. Results were expressed as area of positive staining as a percentage of total background staining. Positively stained percentage area (rather than percentage of cells) was used owing to merging of cell nuclei upon cellular parameter inputting within the ImageJ program. Other easily identifiable cell types known to also express vimentin, such as peri‐cryptal fibroblasts and neuronal cells were visually excluded from ImageJ analysis wherever possible.

### 2.4 Histological assessment of FFPE tissue from stricture resection specimens

A specialist gastrointestinal pathologist (RF) assessed intestinal resection tissue sections from patients included in the study in order to grade the relative levels of fibrosis and inflammation. The pathologist was blinded to the clinical details. Currently, histological scoring systems for intestinal fibrosis are sparse and are not validated. Therefore, both submucosal and subserosal fibrosis were graded in terms of severity and extent, and each was given a nominal score between 0 and 3 based on the proportion of tissue involved. The sum of the submucosal and subserosal fibrosis scores gave a total fibrosis score for each section. Objective assessments of inflammation were also made by scoring ulcers (if present) according to their severity and depth. If more than one ulcer was present, the score of the widest and deepest ulcer was taken forward. Other features that were recorded included the severity of the transmural chronic inflammatory infiltrate (if present) and the presence or absence of serositis.

### 2.5 Laser capture micro‐dissection

Pathology reports for archived intestinal resection specimens from patients with CD were interrogated and blocks containing SCD and NSCD tissue for each patient chosen. Two serial 5 μm sections of FFPE tissue were cut from each block in an RNAse‐free manner onto nuclease‐ and human nucleic acid‐free slides covered with polyethylene naphthalate membrane (Zeiss, Germany). Sections were very briefly counterstained before specific tissue compartments (epithelium, submucosa and muscle) were dissected and collected into collection tubes with adhesive caps (Zeiss, Germany) using the PALM Microbeam Laser Dissector (Zeiss, Germany). Samples were standardised by micro‐dissecting and collecting the same surface areas for each compartment for each slide, to within a 1.5% error: 4 000 000 μm^2^ epithelium, 6 000 000 μm^2^ submucosa, 10 000 000 μm^2^ muscle. Tissue from two serial sections from each block was combined and incubated overnight with proteinase K (10 μL) and Buffer KD (150 μL) prior to total RNA extraction using the miRNeasy FFPE Kit according to the manufacturer's protocol (Qiagen, Hilden, Germany).

### 2.6 RNA extraction, reverse transcription & quantitative real‐time PCR of gene expression

Total RNA, including and excluding small RNAs, was extracted using the miRNeasy and RNeasy kits (Qiagen), respectively. Reverse transcription (RT) and quantitative real time PCR (qPCR) were performed on RNA extracted from intestinal tissue using the High‐Capacity‐RNA to cDNA kit and Taqman assays (Applied Biosystems, Foster City, CA, USA), respectively. MiRNAs extracted from FFPE samples using laser capture micro‐dissection (LCM) underwent RT and qPCR using the miScript system (Qiagen) and a pre‐amplification step owing to low RNA concentrations. Fold changes were calculated using the 2^−δδCT^ method normalised to miR‐26b as an endogenous control for miRNA assays and GAPDH for mRNA assays. The miScript system was used for miRNA extracted from serum without a pre‐amplification step. The synthetic RNA spike‐in, Ce_miR‐39_1, was added to aid data normalisation. Analysis of melt curves was undertaken to ensure a single PCR product was obtained at each reaction. The normalised data were then log10 transformed before statistical analysis. The 7500 Fast System RealTime PCR cycler (Qiagen) was used for all qPCR assays with thermocycling conditions adjusted according to recommendations for each reaction.

The following Taqman probes (Applied Biosystems) were used: miR‐141, Hs04406459_s1; miR‐200a, Hs04231538_s1, miR‐200b, Hs04231483_s1, miR‐200c, Hs04231534_s1; miR‐429, Hs04231584_s1; ZEB1, Hs00232783_m1; ZEB2, Hs00207691_m1; SNAIL, Hs00195591_m1; TWIST, Hs01675818_s1. QPCR was performed on micrornas extracted from intestinal tissue using laser capture microdissection and from serum using the following miScript probes (Qiagen): miR‐26b, MS00003234; miR‐141, MS00003507; miR‐200a, MS00003738; miR‐200b, MS00009016; miR‐200c, MS00003752; miR‐429, MS00004193; Ce_miR_39_1, MS00019789; miR‐451a, MS00004242; miR‐23a, MS00031633.

### 2.7 Statistics

Graphpad Prism analysis software was used to perform Student's *t* tests and Pearson's correlation coefficients as denoted. A *P*‐value <0.05 was considered statistically significant. Experiments were performed in triplicates except for the serum quantifications which were performed in duplicates.

## 3 RESULTS

### 3.1 Patient demographics

The clinical characteristics of patients providing tissue from intestinal resections are shown in Table 1. All patients presented with either ileal (55.6%) or ileocolonic (44.4%) disease, and all had a stricturing phenotype. Table 2 summarises the clinical characteristics of CD patients from whom serum samples were used for miRNA expression profiling. Both the median age at sample collection and disease duration were higher in the SCD group compared to the NSCD group, although this difference was not statistically significant. This is perhaps intuitive, given that the development of fibrosis usually occurs later in the disease course in CD. CDAI scores were almost identical between the two groups (NSCD and SCD). Similarly, there was no significant difference in the proportion of patients prescribed systemic 5‐ASA, antibiotic, thiopurine or anti‐TNFα treatment.

**Table 1:** Clinical characteristics of nine patients with stricturing ileal CD from which intestinal resection specimens were used across immunohistochemistry and miRNA expression analysis studies

Characteristic	Value
Gender	
Male	3 (33.3)
Female	6 (66.7)
Median age at resection (IQR) (y)	33.8 (20)
Median disease duration at resection (IQR) (y)	10.5 (8)
Previous surgery	
Yes	4 (44.4)
No	5 (55.6)
Smoking	
Current	1 (11.1)
Previous	4 (44.4)
Never	4 (44.4)
Disease location	
Ileal	5 (55.6)
Colonic	0 (0.0)
Ileocolonic	4 (44.4)
Upper GI	0 (0.0)
Disease behaviour	
Inflammatory	0 (0.0)
Stricturing	9 (100.0)
Fistulating	0 (0.0)
Oral 5ASA Treatment^a^	
Yes	3 (33.3)
No	6 (66.7)
Oral steroid treatment^a^	
Yes	6 (66.7)
No	3 (33.3)
Thiopurine treatment^a^	
Yes	2 (22.2)
No	7 (77.8)
Methotrexate treatment^a^	
Yes	1 (11.1)
No	8 (88.9)
Infliximab treatment^a^	
Yes	1 (11.1)
No	8 (88.9)
Adalimumab treatment^a^	
Yes	1 (11.1)
No	8 (88.9)
Mean Hb (SD) (g/L)	129.0 (18.8)
Mean WCC (SD) (×10^9^/L)	8.2 (3.7)
Mean CRP (SD) (mg/L)	15.6 (11.8)
Mean albumin (SD) (g/L)	38.8 (9.5)

Unless otherwise stated values represent total numbers, with values in parentheses representing percentage.

IQR, interquartile range; SD, standard deviation; g, grams; mg, milligrams; L, litre.

a Treatment was either current or within the previous 3 mo of surgery.

**Table 2:** Clinical characteristics of patients from whom sera were used for miRNA profiling studies

Characteristic	NSCD group	SCD group	*P* value
Number of patients	29	13	
Gender (%)			
M	13 (44.8)	8 (61.5)	0.323
F	16 (55.2)	5 (38.5)	0.354
Median age (IQR) (y)	32 (17)	45 (28.5)	0.053
Median disease duration (IQR) (y)	4 (6.1)	11 (12.3)	0.24
Disease location (%)			
Ileal	14 (48.3)	6 (46.2)	0.901
Colonic	3 (10.3)	1 (7.6)	0.785
Ileocolonic	12 (41.4)	6 (46.2)	0.774
Upper GI	0 (0.0)	0 (0.0)	1.000
Disease behaviour (%)			
Inflammatory	16 (55.2)	0 (0.0)	0.001
Stricturing	0 (0.0)	13 (100.0)	<0.001
Penetrating	13 (44.8)	0 (0.0)	0.004
Median CDAI score (IQR)	135.5 (135.5)	135 (136.5)	0.59
Smoking status (%)			
Current	6 (20.7)	6 (46.2)	0.095
Previous	2 (6.9)	2 (15.4)	0.392
Never	6 (20.7)	1 (7.6)	0.298
Unknown	15 (51.7)	4 (30.8)	0.214
Stricture present (%)			
Yes	0 (0.0)	11 (84.6)	<0.001
No	29 (100)	2 (15.4)	<0.001
Current treatment^a^ (%)			
5ASA	27 (93.1)	10 (77.0)	0.138
Antibiotics	7 (24.1)	3 (23.1)	0.945
Thiopurines	12 (41.4)	8 (61.5)	0.234
Anti‐TNFα	10 (34.5)	4 (30.8)	0.816

Sera and clinical details were made available from collaborators in Pavia, Italy. An intestinal stricture was present in eleven of the patients in the stricturing phenotype group, with two patients in this group having had a stricture resected previously. Unless otherwise stated, values represent total number, with percentages in brackets.

NSCD, non‐stricturing Crohn's disease; SCD, stricturing Crohn's disease; M, male; F, female; IQR, interquartile range; CDAI, Crohn's disease activity index; 5ASA, 5‐aminosalicylic acid.

a Treatment was either current or within the previous 3 months of sample collection.

### 3.2 Quantified expression patterns of cell‐specific markers are consistent with EMT in fibrotic CD intestinal tissue

**Figure 1:**
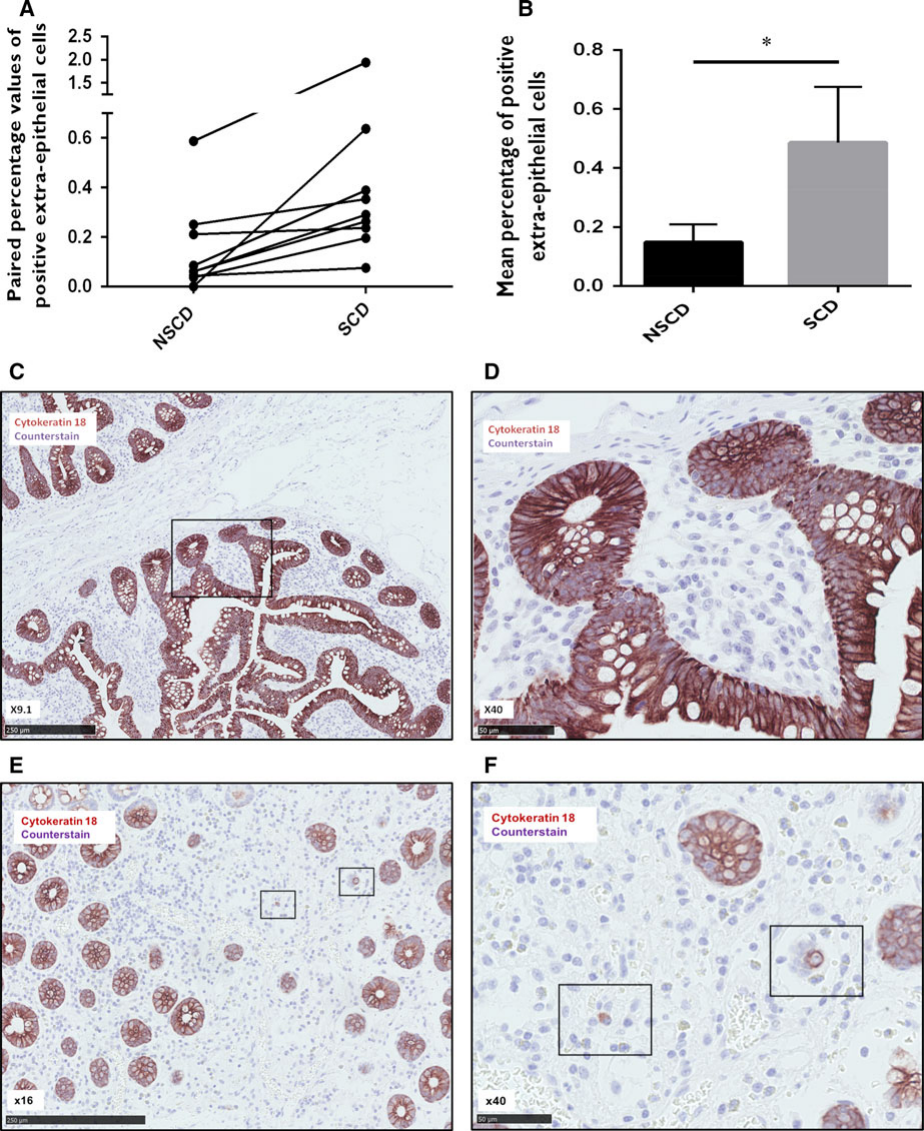
Cytokeratin‐18 immunohistochemical analysis. Nine paired FFPE samples of strictured and non‐strictured intestinal tissue from patients with Crohn's disease (CD) were stained for cytokeratin‐18. Image J was used to analyse the number of extra‐epithelial cells within the lamina propria and submucosa positive for cytokeratin‐18 as a proportion of the total number of cells within 3 randomly selected high power fields per section. Panel A shows the paired percentage values of extra‐epithelial cells positive for cytokeratin‐18 staining in non‐strictured and strictured samples. Panel B shows the mean percentage of extra‐epithelial cells positive for cytokeratin‐18 staining in strictured and non‐strictured tissue. There is a significant increase in the number of positive extra‐epithelial cells in strictured tissue (*P* = 0.043). Bars represent standard error of the mean (SEM). Panels C‐F, representative images of immunohistochemical staining demonstrating specific positivity for cytokeratin‐18 within the epithelium of non‐strictured (C, ×9.1; B, ×40) and strictured (E, ×16; F, ×40) intestinal sections from patients with fibrostenosing CD. Panel D shows the area highlighted in panel C by the black rectangle at a higher power magnification. Panels C and D demonstrate the lack of extra‐epithelial staining of cytokeratin18 in a representative non‐strictured intestinal sample. Panel F shows the area highlighted in panel E by the black rectangle at a higher power magnification. Panels E and F are representative images from strictured intestinal samples demonstrating two positive cellular extra‐epithelial staining patterns of cytokeratin18: membranous and cytoplasmic

**Figure 2:**
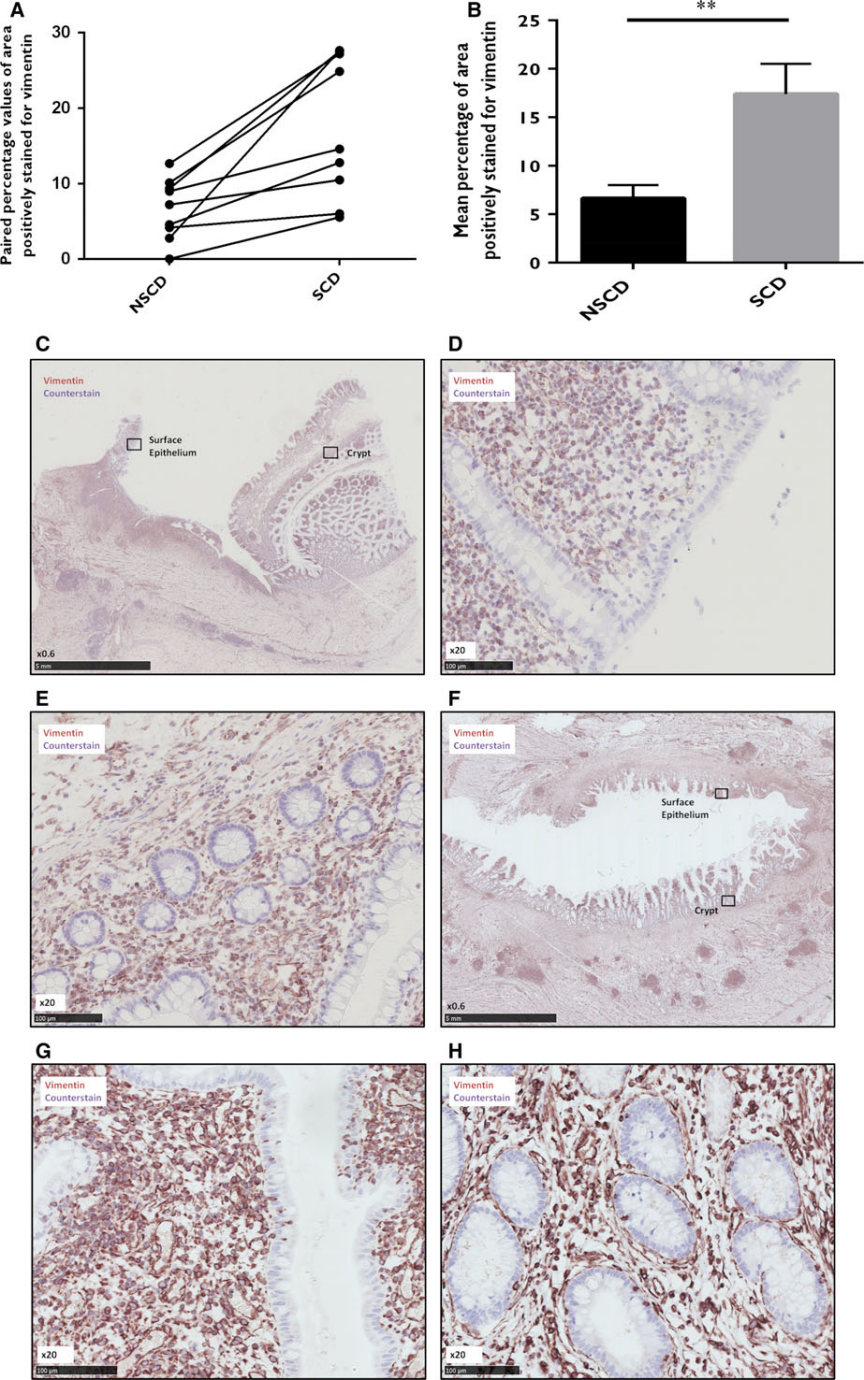
Vimentin immunohistochemical analysis. Nine paired FFPE samples of strictured and non‐strictured intestinal tissue from patients with Crohn's disease (CD) were stained for vimentin. Image J was used to analyse the area (in pixels) positive for vimentin as a proportion of the total area (in pixels) assessed within 2 randomly selected regions of 100 consecutive surface epithelial cells and 3 randomly selected whole crypts in each section of 9 sample pairs. Panel A shows the paired percentage values of area positive for vimentin staining in non‐strictured and strictured samples. Panel B shows the mean percentage of area positive for vimentin staining in strictured and non‐strictured tissue. There is a significant increase in the percentage of area positively stained for vimentin in the strictured specimens compared to the non‐strictured specimens (*P* = 0.003). Bars represent standard error of the mean (SEM). **P < 0.01. Panels C‐H, immunohistochemical staining demonstrating specific positivity for vimentin within the lamina propria and submucosa of non‐strictured (C, ×0.6; D, ×20; E, ×20) and strictured (F, ×0.6; G, ×20; H, ×20) intestinal sections from patients with CD. Panels D and E show the areas of surface epithelium and crypts highlighted in panel C by the black rectangle, respectively, at a higher magnification. Panels G and H show the areas of surface epithelium and crypts highlighted in panel F by the black rectangle, respectively, at a higher magnification. Both the surface epithelium and crypt epithelium show higher positive staining in the strictured specimens than in the non‐strictured specimens

Immunohistochemistry (IHC) staining for the epithelial‐specific cellular marker e‐cadherin revealed a highly specific epithelial pattern in all sections of nine CD surgical sample pairs (Figure S1). However, not a single cell positive for e‐cadherin staining was localised outside of the epithelial architecture (0% for both NSCD and SCD sample groups). Table 3 and Figure 1A and B show the percentages of extra‐epithelial cells positively stained for cytokeratin‐18 as a proportion of total cells stained for each specimen (raw data are shown in Table S2). Positive cellular staining of cytokeratin‐18 within the lamina propria and submucosa was observed in 17 of 18 specimens (94%). The mean percentage of positively stained cells was significantly higher in the SCD group (0.49% vs 0.15%; *P* = 0.043). Representative sample images from IHC staining are shown in Figure 1C‐F. Table 3 and Figure 2A and B show the area percentages positively stained for vimentin as a proportion of epithelial area per field. Raw figures for each section are shown in Table S3. Epithelial expression of the mesenchymal marker vimentin was significantly higher in the SCD group (17.39% vs 6.65%, *P* = 0.003). Representative IHC images are shown in Figure 2C‐H.

**Table 3:** Cytokeratin‐18 and vimentin staining profiles in NSCD and SCD surgical resection specimens

Sample Pair	Cytokeratin‐18 Extra‐epithelial cells positively stained (%)		Vimentin Area positively stained for per slide (%)	
	NSCD	SCD	NSCD	SCD
1	0.59	1.94	0.00	5.53
2	0.25	0.35	7.21	10.49
3	0.00	0.64	12.67	27.16
4	0.08	0.39	4.17	6.00
5	0.21	0.24	4.57	12.78
6	0.04	0.20	9.34	27.51
7	0.04	0.08	2.76	27.62
8	0.06	0.26	10.12	24.83
9	0.06	0.29	8.98	14.58
Mean	0.15	0.49	6.65	17.39
SEM	0.06	0.19	1.35	3.13
Student's *t* test	*P* = 0.043		*P* = 0.003	

For cytokeratin‐18 the ImageJ program was used as described to count the total number of cells and number of positively stained cells in 3 randomly selected fields of ×20 magnification. The sum of the numbers of cells for each field was totalled and a percentage of positive staining cells calculated for each sample as shown below. For vimentin, the ImageJ program was used to calculate the total area (in pixels) assessed and the area positively stained for vimentin (in pixels) in three randomly selected crypts and two randomly selected regions of one hundred consecutive surface epithelial cells at ×20 magnification. The latter was expressed as a percentage of the former. Each data point is an average of percentage values across the three crypts and two surface epithelial areas for each slide. The mean percentages are shown for each group (NSCD vs SCD) and the standard error of the mean (SEM) with the *P* value as calculated using a Student's *t* test showing statistical significance.

### 3.3 Histological tissue scoring allows objective assessment of fibrosis and inflammation and correlation with EMT marker expression

As expected, all blocks containing stricture had significantly higher total fibrosis scores than blocks containing no stricture (9.55 vs 5.11, *P* = 0.003; Table 4, Table S4). The mild to moderate scores for NSCD blocks confirmed the field change effect of histological fibrosis progression (Table S4). No dysplasia was found in any section. Total ulcer scores, used as correlates for inflammation, were not significantly different between the two groups (*P* = 0.7; Table 4).

**Table 4:** Total histological fibrosis and ulcer scores

	Mean total fibrosis score	SE	Mean total ulcer score	SE
NSCD	5.11	1.03	1.77	0.79
SCD	9.55	0.53	2.22	0.89
*P* value	0.003		0.70	

Nine sample sets of archived small intestinal specimens from patients with CD were graded by an independent gastrointestinal histopathologist who was blinded to clinicopathological details and given total fibrosis and total ulcer scores. The mean total fibrosis scores were significantly higher in the stricture sets than the non‐stricture sets, with no significant difference in the total ulcer scores between the two groups.

This scoring system allowed the evaluation of correlations between IHC scores and grades of fibrosis. Values of staining scores of both cytokeratin‐18 and vimentin were plotted against corresponding values of fibrosis scores. No correlation between vimentin scores and composite fibrosis scores was demonstrated but a positive correlation was observed between cytokeratin‐18 IHC scores and extent of subserosal fibrosis (*R* ^*2*^ **=* *0.66, *P* < 0.001, Figure S2).

### 3.4 The mir‐200 family is down‐regulated in fibrotic CD intestinal tissue and specifically within the epithelium

**Figure 3:**
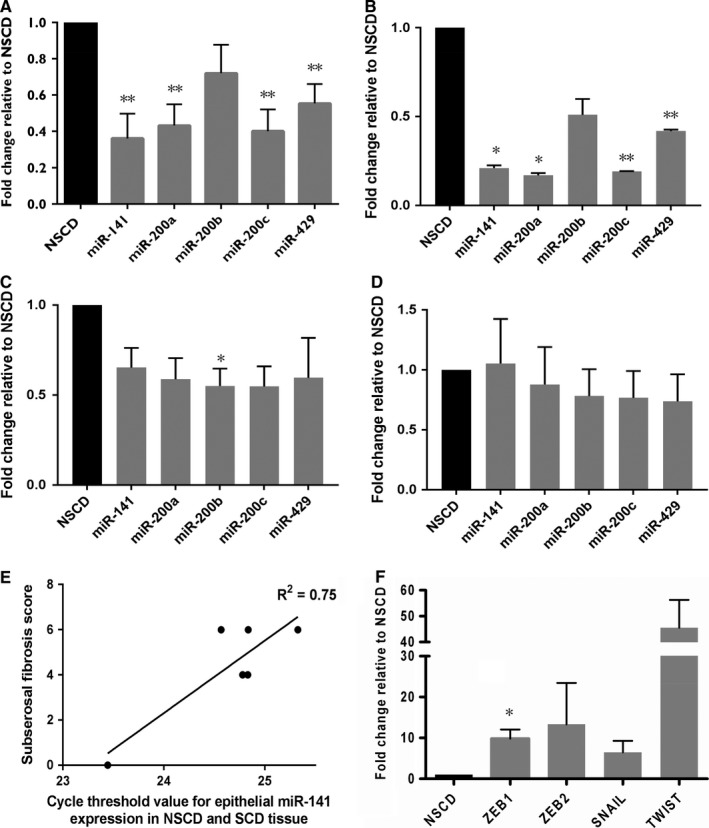
MiR‐200 family and EMT transcription factor expression in strictured and non‐strictured Crohns disease small intestine. Panel A, MicroRNA‐200 family expression assessed using RT‐qPCR on RNA extracted from eight matched paired NSCD (black) and SCD (grey) tissue samples. Fold changes of normalised expression of each of the five members of the miR‐200 family in SCD samples compared to NSCD samples is shown. Fold changes were calculated using the 2^−δδCT^ method. Four of the five members of the miR‐200 family were significantly down‐regulated: miR‐141 (*P* = 0.0022), miR‐200a (*P* = 0.0017), miR‐200c (*P* = 0.0015) and miR‐429 (*P* = 0.0042). Bars represent mean values with standard error. **P < 0.01. Panel B‐D, MiR‐200 family expression by tissue compartment. Panel B shows fold change of normalised expression of each of the five members of the miR‐200 family in the epithelial compartment of SCD samples compared to NSCD samples. Four of the five members of the miR‐200 family were significantly down‐regulated: miR‐141 (*P* = 0.0151), miR‐200a (*P* = 0.01310), miR‐200c (*P* = 0.0015) and miR‐429 (*P* = 0.0080). Panel C shows fold change of normalised expression of each of the five members of the miR‐200 family in the submucosal compartment of SCD samples compared to NSCD samples. MiR‐200b was found to be significantly down‐regulated in SCD samples in this compartment (*P* = 0.0432). No other member of the miR‐200 family was significantly differentially expressed. There was no significant difference found in the expression of any member of the miR‐200 family within the smooth muscle compartment (D). Fold changes were calculated using the 2^−δδCT^ method. Bars represent mean values with standard error. * *P* < 0.05, ** *P* < 0.01. Panel E, correlation between subserosal fibrosis score and epithelial expression of miR‐141 in CD small intestinal tissue. The combination of subserosal fibrosis extent and severity (the subserosal fibrosis score) as assigned by an independent expert gastrointestinal histopathologist blinded to clinicopathological details are plotted against the Ct values of miR‐141 in six samples of SCD and NSCD intestinal tissue. A positive correlation is observed (*R*² = 0.75, *P* = 0.02). Panel F, EMT transcription factor expression. RT‐qPCR was performed on RNA extracted from eight matched sample pairs of strictured (grey bars) and non‐strictured (black bar) small intestinal tissue from patients with fibrostenosing CD. Fold changes were calculated using the 2^−δδCT^ method normalised to *GAPDH*. *ZEB1* was found to be significantly up‐regulated (*P* = 0.035). Three other transcription factors were not significantly up‐regulated: *ZEB2* (*P* = 0.259), *SNAIL* (*P* = 0.101), *TWIST* (*P* = 0.173). Bars represent mean standard error. *P < 0.05

In conjunction with IHC evidence of EMT occurring in association with fibrosis in intestinal resection specimens from patients with CD, we also sought to measure expression of the miR‐200 family in eight paired NSCD and SCD samples of whole tissue. The expression of all five members of the miR‐200 family was measured using RT‐qPCR and normalised to the expression of miR‐26b as the control miRNA. Four of the five members of the miR‐200 family were found to be down‐regulated in SCD tissue compared to NSCD tissue: miR‐141 (*P* = 0.0022), miR‐200a (*P* = 0.0017), miR‐200c (*P* = 0.0015) and miR‐429 (*P* = 0.0042). MiR‐200b expression also displayed a trend towards down‐regulation in SCD tissue, although significance was not reached (*P* = 0.1166) (Figure 3A).

Given the apparent regulatory role of the miR‐200 family in EMT,^31^, ^32^, ^33^, ^35^ we next sought to measure expression in different intestinal tissue compartments in an independent cohort of SCD and NSCD controls (n = 3 sample pairs). LCM was used to isolate regions of epithelium and submucosa as well as smooth muscle as a control compartment from each tissue section (Figure S3). As predicted RNA yield was low, so standardisation of RNA input involved dissecting a consistent area per tissue compartment (Table S5 shows the areas harvested for each sample; Table S6 shows total area grouped by NSCD and SCD samples). The expression data were also normalised to miR‐16 to facilitate comparison of relative expression levels. Figure 3B‐D shows the expression of members of the miR‐200 family in the epithelial, submucosal and smooth muscle compartments of paired NSCD and SCD samples. Significant down‐regulation of four of the five members of the miR‐200 family was observed within the epithelium (Figure 3B) of SCD samples compared to NSCD samples (miR‐141 fold change 0.21, *P* = 0.015; miR‐200a fold change 0.17, *P* = 0.013; miR‐200b fold change 0.51, *P* = 0.090; miR‐200c fold change 0.19, *P* = 0.002; miR‐429 fold change 0.42, *P* = 0.008). Within the submucosal compartment (Figure 3C), miR‐200b was found to be significantly down‐regulated in SCD samples compared to NSCD samples (fold change 0.55; *P* = 0.0432), whereas the other four members were not (miR‐141 fold change 0.65, *P* = 0.087; miR‐200a fold change 0.59, *P* = 0.073; miR‐200c fold change 0.55, *P* = 0.056; miR‐429 fold change 0.60, *P* = 0.21). No significant difference in expression of any miR‐200 family member was observed in the smooth muscle compartment (Figure 3D) between NSCD and SCD samples (miR‐141 fold change 1.05, *P* = 0.900; miR‐200a fold change 0.88, *P* = 0.735; miR‐200b fold change 0.78, *P* = 0.433; miR‐200c fold change 0.77, *P* = 0.408; miR‐429 fold change 0.74, *P* = 0.365).

### 3.5 Epithelial down‐regulation of miR‐141 is associated with the histological degree of fibrosis in intestinal CD samples

The histological scoring system allowed a comparison of the fibrosis grade to the compartmental expression of the miR‐200 family in the cohort of SCD patients used for the LCM study. A negative correlation was observed between epithelial miR‐141 expression and subserosal fibrosis score (n = 3 sample pairs, *R* ^2^ = 0.75, *P* = 0.02, Figure 3E). Increased cycle threshold values (and therefore reduced expression) of miR‐141 appeared to be associated with higher subserosal fibrosis scores, which were composite scores of both the extent and severity of subserosal fibrosis. No significant correlation was observed between the compartmental expression of any other miRNA and histological fibrosis score parameter (Table S7).

### 3.6 Known EMT effectors and downstream targets of the miR‐200 family are dysregulated in fibrotic CD intestinal tissue

The expression of transcriptional effectors of EMT and known downstream targets of miR‐141 and the other members of the miR‐200 family was measured in the same cohort of eight matched sample pairs in which miR‐200 down‐regulation had been demonstrated (Figure 3F). A significant body of evidence links a group of transcription factors to the control of EMT by the miR‐200 family, including *ZEB1*,* ZEB2*,* SNAIL* and *TWIST*.^37^, ^39^, ^40^ Using RT‐qPCR one such transcription factor, *ZEB1*, was found to be significantly up‐regulated in strictured tissue (*P* = 0.035) (Figure 3F). Another three transcription factors were found to be up‐regulated but not significantly so: *ZEB2* (*P* = 0.259), *SNAIL* (*P* = 0.101), *TWIST* (*P* = 0.173) (Figure 3F). Given that miRNAs are negative gene regulators, this up‐regulation of the downstream targets is in keeping with a functionally relevant miRNA down‐regulation in fibrotic tissue.

### 3.7 The miR‐200 family is lowly expressed in sera of CD patients and profiles do not differ between disease phenotypes

**Figure 4:**
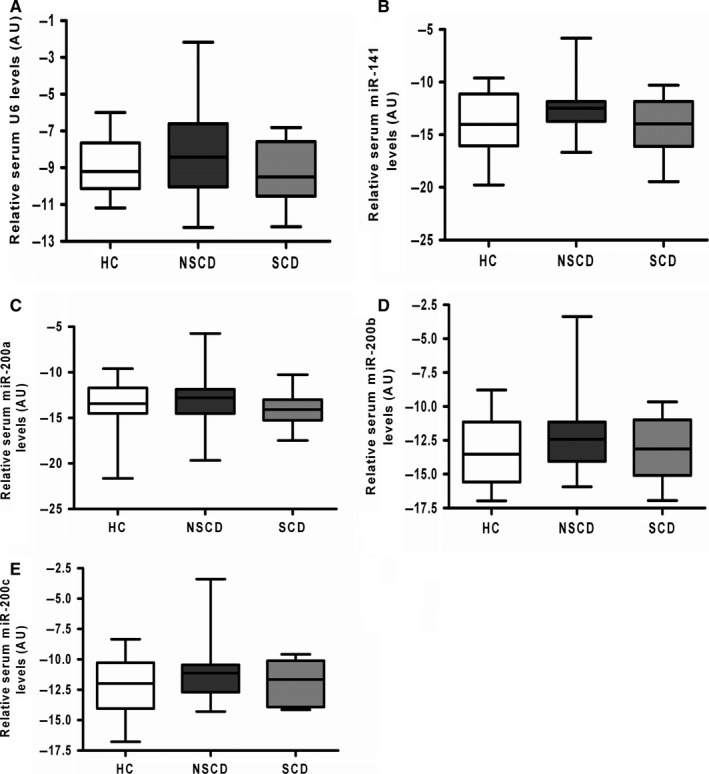
Serum expression profiles of the miR‐200 family in patients with Crohn's disease and healthy controls. MiRNAs were extracted from sera from patients with stricturing CD (SCD; n = 10), non‐stricturing CD (NSCD; n = 23) and healthy controls (HC; n = 12). Box and whisker plots demonstrate relative expression of U6 (A), miR‐141 (B), miR‐200a (C), miR‐200b (D) and miR‐200c (E). One way ANOVA testing revealed no significant differences between the clinical groups for any member of the miR‐200 family nor the control U6. Mir‐429 was excluded from this analysis as correlation between duplicate readings was poor. AU, arbitrary units; HC, healthy controls; NSCD, non‐stricturing Crohn's disease; SCD, stricturing Crohn's disease

The observation that the miR‐200 family is down‐regulated within the intestinal epithelium in SCD is potentially clinically relevant for the development of biomarkers of intestinal fibrosis in CD, in that the epithelium is readily sampled by endoscopic mucosal biopsy. However, non‐invasive testing is clearly more desirable given the potential risks with endoscopic procedures and the potential inaccessibility of small intestinal lesions. Therefore, given that serum miRNA profiles can be stable over time and assayed successfully,^31^, ^37^, ^41^ the expression of the miR‐200 family in the sera of patients with CD was studied, with the aim to assess whether alterations in levels in patients with SCD could be found. An independent cohort of 42 CD patients (Table 2) was used to assess whether alterations in miR‐200 family levels could be identified in the serum of SCD patients. Serum samples with significant degrees of haemolysis were excluded from further analysis (Figure S4). No significant differences in expression of the control gene U6 (*P* = 0.2639), miR‐141 (*P* = 0.1474), miR‐200a (*P* = 0.5941), miR‐200b (*P* = 0.3249) or miR‐200c (*P* = 0.3749) were observed between the clinical groupings (one‐way analysis of variance; Figure 4).

## 4 DISCUSSION

Although published data suggest a contribution of EMT to the development of intestinal fibrosis in mouse models,^30^ there are only very few reports of cellular EMT events in human CD.^31^, ^32^, ^37^ This is mainly due to the inherent difficulties associated with such studies: well‐phenotyped *ex vivo* samples are necessary and most commercially available human intestinal cell lines are from malignant tumours. Here, these limitations were addressed by using well‐characterised samples from clearly defined CD patient groups.

IHC revealed no extra‐epithelial staining of e‐cadherin in any surgical specimen (SCD or NSCD). However, the cytokeratin‐18 and vimentin profiles lend support for EMT in CD samples. This possible early loss of e‐cadherin during EMT in human sections suggests a potential difference in staining patterns between published animal model data and well characterised human intestinal tissue. E‐cadherin is important for cellular adhesion^9^ and the early loss of this transmembrane protein might be a necessary initial step for subsequent EMT. This finding should prompt the use of a range of epithelial markers (eg cytokeratin‐8, ‐17, ‐19 and EMA) instead of solely e‐cadherin when working with human material.

The absolute percentages of extra‐epithelial cells positive for cytokeratin‐18 were very low, perhaps because cytokeratin‐18 is also lost relatively early in EMT. Given that tissue used in this study was taken from clinically relevant strictures, it is likely that fibrosis was at a late stage of development. It is also possible therefore that fully transitioned cells will have been missed, or that the contribution of EMT at this stage is less than at the initiatory stages of fibrosis. In contrast, the epithelial vimentin staining percentage values were relatively high, potentially representing a population of cells primed for transitioning. It is possible that some of these positively stained cells represented intra‐epithelial lymphocytes, although both NSCD and SCD groups would have been affected by this to a similar degree given that the histological inflammation scores were not significantly different between the two groups. The acquisition of mesenchymal markers, therefore, may occur earlier in EMT than purported, and certainly earlier than in animal models.^30^ These findings challenge the traditional view of EMT as a linear progression of cells losing epithelial characteristics before transitioning into mesenchymal cells. Furthermore, the morphology of extra‐epithelial cells positive for cytokeratin‐18 were neither of classical mesenchymal nor epithelial appearance, in keeping with the theory of “partial EMT”.^29^ Indeed, this intermediate state of transitioning cell is thought to occur more frequently in fibrosis‐related EMT than cancer‐related EMT.^27^, ^29^

The significant alterations in EMT cellular marker expression between NSCD and SCD tissue occurred in sample groups between which objective inflammatory scores were not significantly different. This contrasts with previous data showing reduced e‐cadherin expression and increased vimentin expression in inflamed colonic mucosal biopsy samples from patients with IBD.^36^ It is unclear whether the specimens in that study also had evidence of fibrosis, although this would seem less likely given that patients with UC were included and all samples were colonic, rather than ileal, in origin. In addition, the samples in the present study were chosen carefully to avoid including dysplasia or established cancer, given the well‐characterised association of EMT with gastrointestinal cancer progression. Therefore, it can be assumed that the EMT marker alterations demonstrated here were not being driven by malignancy or inflammation.

The histological fibrosis scoring system used here was not validated on an external cohort, nor by a second pathologist. Despite these limitations, a significant correlation was observed between cytokeratin‐18 expression and the degree of subserosal fibrosis. This would seem counterintuitive given that, at a tissue level, the epithelial origin of transitioning cells and the serosa are some distance apart. It is possible, therefore, that the contribution of EMT to intestinal fibrosis is minimal or that it occurs in ways other than the delivery of active matrix‐producing cells. In models of renal fibrosis, injured epithelial cells have been found to undergo partial EMT but remain close to the tubular epithelium and secrete cytokines and exosomes that aid fibrocyte recruitment and macrophage stimulation.^27^, ^28^, ^42^ It is possible that a similar paradigm exists in the gut, whereby epithelial cells undergoing partial EMT modulate fibrosis in the interstitium by paracrine, rather than migratory and matrix‐producing, functions. The extra‐epithelial cells identified by cytokeratin‐18 staining could therefore represent “stranded” epithelial cells following end‐stage fibrosis, “partial” EMT cells effecting fibrosis through paracrine channels or “migrant” cells invading deeper tissue layers for matrix deposition. Further work is required to clarify this.

Dysregulation of miRNA‐200 family members was shown to occur not only between NSCD and SCD tissue, but within the epithelial layer specifically. This is a novel finding, and one which makes biological sense as the altered miRNA expression is juxtaposed to the origin of transitioning cells. Given the relatively small sample size, this finding should be explored in larger throughput studies. In addition, significant negative correlation was found between miR‐141 expression and the subserosal fibrosis score. Hence, alterations in molecular expression within the superficial mucosa may reflect cellular events occurring within deeper intestinal layers in CD, evidence for which has been presented before.^43^ Conversely, no correlation was found between the remaining members of the miR‐200 family and the other parameters of histological fibrosis. This relatively limited correlation may be due to the fact that alterations in miRNA levels may be only early events in the process of EMT, or simply a function of the small sample size.

The up‐regulation in *ZEB1* expression in SCD samples makes biological sense given that miRNAs are negative gene regulators. The principal gene target of *ZEB1* is *CDH1*, which codes for e‐cadherin. Loss of e‐cadherin and subsequent cellular adhesion is a hallmark of EMT 16, 29, 44 and this may occur under the control of the miR‐200 family via the transcription factors *ZEB1* and perhaps also *ZEB2, SNAIL* and *TWIST;* this loss may be an “EMT trigger” in this setting. Further work is clearly required to demonstrate a functional link between the miR‐200 family and control of type 2 EMT in the intestine.

If this mechanism was supported by further studies, potential therapeutic strategies may involve miRNA manipulation. For example, recent work has demonstrated that microvesicle‐delivered miR‐200b reduces colonic fibrosis in TNBS‐induced experimental colitis.^45^ Furthermore, preclinical trials that assess the therapeutic impact of targeting miRNAs have already begun.^46^, ^47^ Limitations to therapeutic use of miRNA modulation, include off‐target effects^48^, ^49^, ^50^ and drug delivery, which is a particular problem when dealing with the gastrointestinal tract.

Serum expression of the miR‐200 family did not differ between healthy controls, NSCD or SCD, possibly due to an overlap of fibrotic burden or generally low miR‐200 expression levels. The miR‐200 family is likely to be functionally relevant in CD fibrosis, but does not appear to be amenable to non‐invasive serum sampling for assessment of fibrotic burden or monitoring therapeutic intervention. The focus of future research therefore should be on improving the understanding of the processes underpinning intestinal fibrosis and identifying potential therapeutic targets.

In conclusion, our results indicate the occurrence of EMT in fibrotic intestinal CD tissue, and that this occurs independently of the effect of inflammation. Additional work is now required to confirm these findings using a wide range of epithelial and mesenchymal markers. The miR‐200 family, known regulators of EMT in other organs, are dysregulated in fibrotic CD intestine, and this dysregulation is specific to the epithelium. Functional studies, together with further larger scale studies using similarly well‐phenotyped samples are warranted.

## CONFLICTS OF INTEREST

The authors confirm that there are no conflicts of interest.

## References

[1] Munkholm P , Langholz E , Davidsen M . Disease activity courses in a regional cohort of Crohn's disease patients. Scand J Gastroenterol. 2009;30:699‐706. 10.3109/00365529509096316 7481535

[2] Nguyen GC , Nugent Z , Shaw S , Bernstein CN . Outcomes of patients with Crohn's disease improved from 1988 to 2008 and were associated with increased specialist care. Gastroenterology. 2011;141:90‐97. 21458455 10.1053/j.gastro.2011.03.050

[3] van der Valk ME , Mangen MJ , Leenders M , et al. Healthcare costs of inflammatory bowel disease have shifted from hospitalisation and surgery towards anti‐TNFα therapy: results from the COIN study. Gut. 2014;63:72‐79. 23135759 10.1136/gutjnl-2012-303376

[4] Feagan BG , Bala M , Yan S , et al. Unemployment and disability in patients with moderately to severely active Crohn's disease. J Clin Gastroenterol. 2005;39:390‐395. 15815207 10.1097/01.mcg.0000159220.70290.41

[5] Jeuring SF , van den Heuvel TR , Liu LY , et al. Improvements in the long‐term outcome of Crohn's disease over the past two decades and the relation to changes in medical management: results from the population‐Based IBDSL cohort. Am J Gastroenterol. 2017;112:325‐336. 27922024 10.1038/ajg.2016.524

[6] Lawrance IC , Maxwell L , Doe W . Altered response of intestinal mucosal fibroblasts to profibrogenic cytokines in inflammatory bowel disease. Inflamm Bowel Dis. 2001;7:226‐236. 11515849 10.1097/00054725-200108000-00008

[7] Mifflin RC , Pinchuk IV , Saada JI , et al. Intestinal myofibroblasts: targets for stem cell therapy. Am J Physiol Gastrointest Liver Physiol. 2011;300:G684‐G696. 21252048 10.1152/ajpgi.00474.2010 PMC3094146

[8] Grgic I , Duffield JS , Humphreys BD . The origin of interstitial myofibroblasts in chronic kidney disease. Pediatr Nephrol. 2012;27:183‐193. 21311912 10.1007/s00467-011-1772-6 PMC3116994

[9] Mehta S , Nijhuis A , Kumagai T , et al. Defects in the adherens junction complex (E‐cadherin/ β‐catenin) in inflammatory bowel disease. Cell Tissue Res. 2015;360:749‐760. 25238996 10.1007/s00441-014-1994-6

[10] Qin Y , Capaldo C , Gumbiner BM , Macara IG . The mammalian Scribble polarity protein regulates epithelial cell adhesion and migration through E‐cadherin. J Cell Biol. 2005;171:1061‐1071. 16344308 10.1083/jcb.200506094 PMC2171311

[11] Whiteman EL , Liu CJ , Fearon ER , Margolis B . The transcription factor snail represses Crumbs3 expression and disrupts apico‐basal polarity complexes. Oncogene. 2008;27:3875‐3879. 18246119 10.1038/onc.2008.9 PMC2533733

[12] Weinberg RA . Twisted epithelial‐mesenchymal transition blocks senescence. Nat Cell Biol. 2008;10(9):1021‐1023. 18758491 10.1038/ncb0908-1021

[13] Lamouille S , Xu J , Derynck R . Molecular mechanisms of epithelial‐mesenchymal transition. Nat Rev Mol Cell Biol. 2014;15:178‐196. 24556840 10.1038/nrm3758 PMC4240281

[14] Stone RC , Pastar I , Ojeh N , et al. Epithelial‐mesenchymal transition in tissue repair and fibrosis. Cell Tissue Res. 2016;365:495‐506. 27461257 10.1007/s00441-016-2464-0 PMC5011038

[15] Humphreys BD , Lin SL , Kobayashi A , et al. Fate tracing reveals the pericyte and not epithelial origin of myofibroblasts in kidney fibrosis. Am J Pathol. 2010;176:85‐97. 20008127 10.2353/ajpath.2010.090517 PMC2797872

[16] Iwano M , Plieth D , Danoff TM , et al. Evidence that fibroblasts derive from epithelium during tissue fibrosis. J Clin Invest. 2002;110:341‐350. 12163453 10.1172/JCI15518 PMC151091

[17] Rastaldi MP , Ferrario F , Giardino L , et al. Epithelial‐mesenchymal transition of tubular epithelial cells in human renal biopsies. Kidney Int. 2002;62:137‐146. 12081572 10.1046/j.1523-1755.2002.00430.x

[18] Sun YB , Qu X , Caruana G , Li J . The origin of renal fibroblasts/myofibroblasts and the signals that trigger fibrosis. Differentiation. 2016;92:102‐107. 27262400 10.1016/j.diff.2016.05.008

[19] Zeisberg M , Duffield JS . Resolved: EMT produces fibroblasts in the kidney. J Am Soc Nephrol. 2010;21:1247‐1253. 20651165 10.1681/ASN.2010060616

[20] Diaz R , Kim JW , Hui JJ , et al. Evidence for the epithelial to mesenchymal transition in biliary atresia fibrosis. Hum Pathol. 2008;39:102‐115. 17900655 10.1016/j.humpath.2007.05.021

[21] Rygiel KA , Robertson H , Marshall HL , et al. Epithelial‐mesenchymal transition contributes to portal tract fibrogenesis during human chronic liver disease. Lab Invest. 2008;88:120‐123. 10.1038/labinvest.3700704 18059363

[22] Tennakoon AH , Izawa T , Wijesundera KK , et al. Analysis of glial fibrillary acidic protein (GFAP)‐expressing ductular cells in a rat liver cirrhosis model induced by repeated injections of thioacetamide (TAA). Exp Mol Pathol. 2015;98:476‐485. 25758201 10.1016/j.yexmp.2015.03.010

[23] Chen LJ , Ye H , Zhang Q , et al. Bleomycin induced epithelial‐mesenchymal transition (EMT) in pleural mesothelial cells. Toxicol Appl Pharmacol. 2015;283:75‐82. 25595642 10.1016/j.taap.2015.01.004

[24] Zolak JS , Jagirdar R , Surolia R , et al. Pleural mesothelial cell differentiation and invasion in fibrogenic lung injury. Am J Pathol. 2013;182:1239‐1247. 23399488 10.1016/j.ajpath.2012.12.030 PMC3620419

[25] Di Meglio F , Castaldo C , Nurzynska D , et al. Epithelial‐mesenchymal transition of epicardial mesothelium is a source of cardiac CD117‐positive stem cells in adult human heart. J Mol Cell Cardiol. 2010;49:719‐727. 20566360 10.1016/j.yjmcc.2010.05.013

[26] Limana F , Zacheo A , Mocini D , et al. Identification of myocardial and vascular precursor cells in human and mouse epicardium. Circ Res. 2017;101:1255‐1265. 10.1161/CIRCRESAHA.107.150755 17947800

[27] Grande MT , Sánchez‐Laorden B , López‐Blau C , et al. Snail1‐induced partial epithelial‐to‐mesenchymal transition drives renal fibrosis in mice and can be targeted to reverse established disease. Nat Med. 2015;21:989‐997. 26236989 10.1038/nm.3901

[28] Lovisa S , LeBleu VS , Tampe B , et al. Epithelial‐to‐mesenchymal transition induces cell cycle arrest and parenchymal damage in renal fibrosis. Nat Med. 2013;21(9):998‐1009. 10.1038/nm.3902 PMC4587560 26236991

[29] Nieto MA , Huang RY , Jackson RA , Thiery JPEMT . Cell. 2016;2016(166):21‐45. 10.1016/j.cell.2016.06.028 27368099

[30] Flier SN , Tanjore H , Kokkotou EG , et al. Identification of epithelial to mesenchymal transition as a novel source of fibroblasts in intestinal fibrosis. J Biol Chem. 2010;285:20202‐20212. 20363741 10.1074/jbc.M110.102012 PMC2888433

[31] Scharl M , Huber N , Lang S , et al. Hallmarks of epithelial to mesenchymal transition are detectable in Crohn's disease associated intestinal fibrosis. Clinical and Translational Medicine. 2015;4:1. 25852817 10.1186/s40169-015-0046-5 PMC4384762

[32] Chen X , Liang H , Zhang J , et al. Secreted microRNAs: a new form of intercellular communication. Trends Cell Biol. 2012;22:125‐132. 22260888 10.1016/j.tcb.2011.12.001

[33] Díaz‐Martín JI , Díaz‐López A , Moreno‐Bueno G , et al. A core microRNA signature associated with inducers of the epithelial‐to‐mesenchymal transition. J Pathol. 2014;232:319‐329. 24122292 10.1002/path.4289

[34] Engelsvold DH , Utheim TP , Olstad OK , et al. MiRNA and mRNA expression profiling identifies members of the miR‐200 family as potential regulators of epithelial‐mesenchymal transition in pterygium. Exp Eye Res. 2013;115:189‐198. 23872359 10.1016/j.exer.2013.07.003 PMC4278354

[35] Gregory PA , Bert AG , Paterson EL , et al. The miR‐200 family and miR‐205 regulate epithelial to mesenchymal transition by targeting ZEB1 and SIP1. Nat Cell Biol. 2008;10:593‐601. 18376396 10.1038/ncb1722

[36] Chen Y , Xiao Y , Ge W , et al. miR‐200b inhibits TGF‐β1‐induced epithelial‐mesenchymal transition and promotes growth of intestinal epithelial cells. Cell Death Dis. 2013;4:e541. 23492772 10.1038/cddis.2013.22 PMC3613822

[37] Zidar N , Boštjančič E , Jerala M , et al. Down‐regulation of microRNAs of the miR‐200 family and up‐regulation of Snail and Slug in inflammatory bowel diseases ‐ hallmark of epithelial‐mesenchymal transition. J Cell Mol Med. 2016;20:1813‐1820. 27113480 10.1111/jcmm.12869 PMC5020622

[38] Lewis A , Mehta S , Hanna LN , et al. Low Serum Levels of microrna‐19 Are Associated with a Stricturing Crohn's Disease Phenotype. Inflamm Bowel Dis. 2015;21:1926‐1934. 25985247 10.1097/MIB.0000000000000443

[39] Liu YN , Yin JJ , Abou‐Kheir W , et al. MiR‐1 and miR‐200 inhibit EMT via Slug‐ dependent and tumorigenesis via Slug‐independent mechanisms. Oncogene. 2013;32:296‐306. 22370643 10.1038/onc.2012.58 PMC7580497

[40] Wang B , Koh P , Winbanks C , et al. MiR‐200a Prevents renal fibrogenesis through repression of TGF‐β2 expression. Diabetes. 2014;60:280‐287. 10.2337/db10-0892 PMC3012183 20952520

[41] Nijhuis A , Biancheri P , Lewis A , et al. In Crohn's disease fibrosis‐reduced expression of the miR‐29 family enhances collagen expression in intestinal fibroblasts. Clin Sci (Lond). 2014;127:341‐350. 24641356 10.1042/CS20140048

[42] Borges FT , Melo SA , Ozdemir BC , et al. TGF‐b1‐containing exosomes from injured epithelial cells activate fibroblasts to initiate tissue regenerative responses and fibrosis. J Am Soc Nephrol. 2013;24:385‐392. 23274427 10.1681/ASN.2012101031 PMC3582210

[43] Di Sabatino A , Jackson CL , Pickard KM , et al. Transforming growth factor β signalling and matrix metalloproteinases in the mucosa overlying Crohn's disease strictures. Gut. 2009;58:777‐789. 19201776 10.1136/gut.2008.149096

[44] Thiery JP . Epithelial‐mesenchymal transitions in development and pathologies. Curr Opin Cell Biol. 2003;15:740‐746. 14644200 10.1016/j.ceb.2003.10.006

[45] Yang J , Zhou CZ , Zhu R , et al. miR‐200b‐containing microvesicles attenuate experimental colitis associated intestinal fibrosis by inhibiting the development of EMT. J Gastroenterol Hepatol. 2017;32:1966‐1974. 28370348 10.1111/jgh.13797

[46] Bouchie A . First microRNA mimic enters clinic. Nat Biotechnol. 2013;31:577. 23839128 10.1038/nbt0713-577

[47] Janssen HL , Reesink HW , Lawitz EJ , et al. Treatment of HCV infection by targeting microRNA. N Engl J Med. 2013;368:1685‐1694. 23534542 10.1056/NEJMoa1209026

[48] Jackson A , Linsley PS . The therapeutic potential of microRNA modulation. Discov Med. 2010;9:311‐318. 20423675

[49] Lewis A , Nijhuis A , Mehta S , et al. Intestinal fibrosis in Crohn's disease: role of microRNAs as fibrogenic modulators, serum biomarkers, and therapeutic targets. Inflamm Bowel Dis. 2015;21:1141‐1150. 25636122 10.1097/MIB.0000000000000298

[50] Pottier N , Cauffiez C , Perrais M , et al. FibromiRs: translating molecular discoveries into new anti‐fibrotic drugs. Trends Pharmacol Sci. 2014;35:119‐126. 24560301 10.1016/j.tips.2014.01.003

