# Supplementary material for: Epithelial down‐regulation of the miR‐200 family in fibrostenosing Crohn's disease is associated with features of epithelial to mesenchymal transition
Source: J Cell Mol Med. 2018 Sep 6;22(11):5617–28. doi: 10.1111/jcmm.13836 (PMC6201355; doi:10.1111/jcmm.13836)
Supplement: Supplementary file 1 [file JCMM-22-5617-s001.docx]

**Supplementary Figures**

**A B**

**
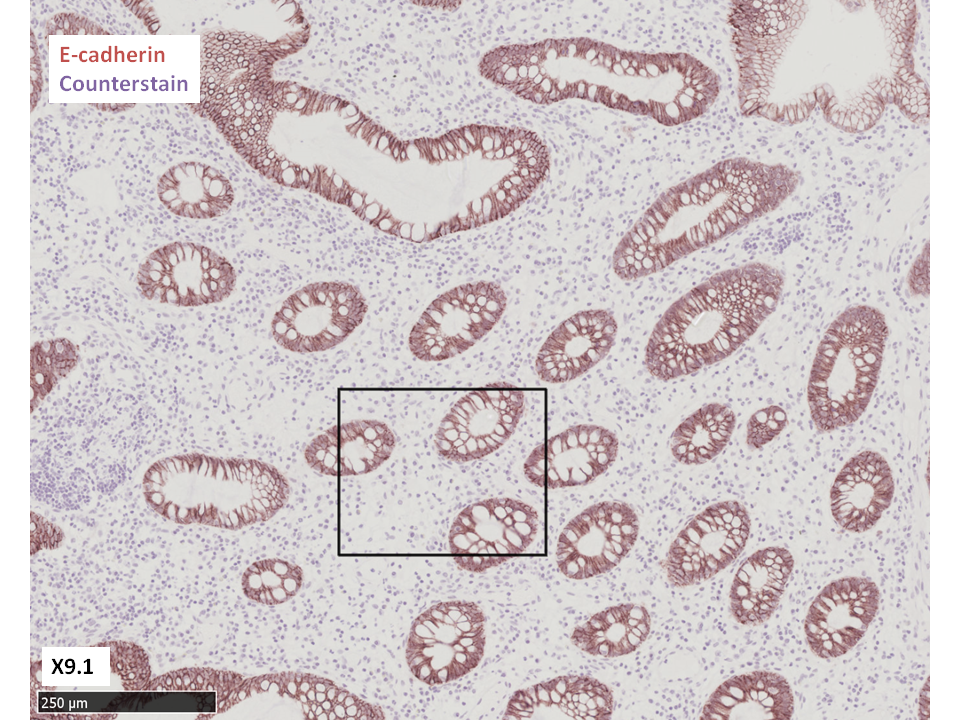

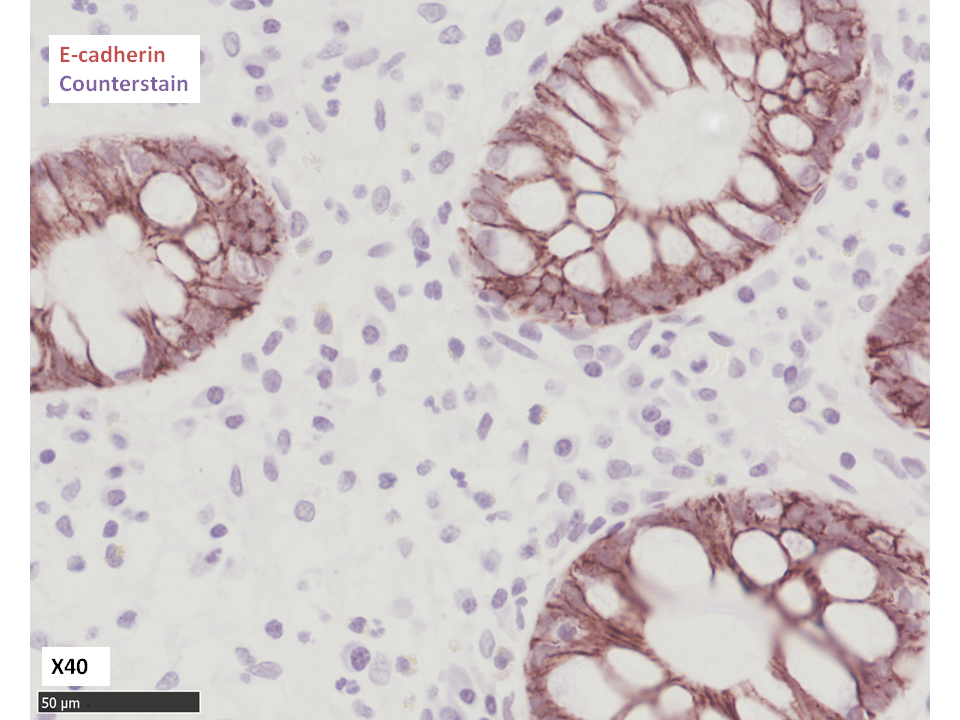
**

**C D**

**
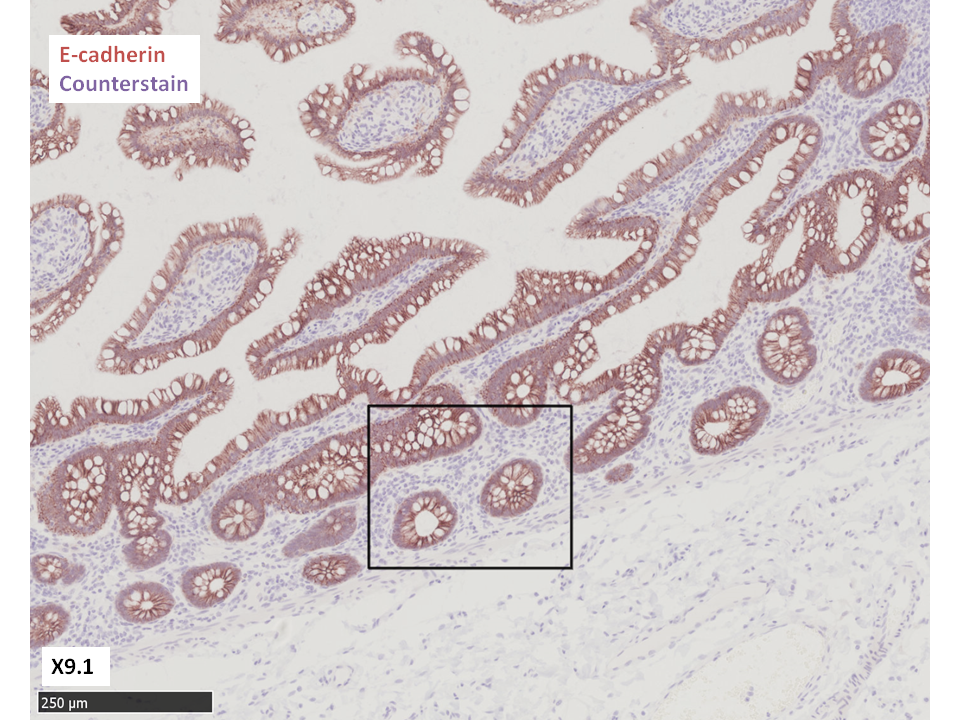
** *
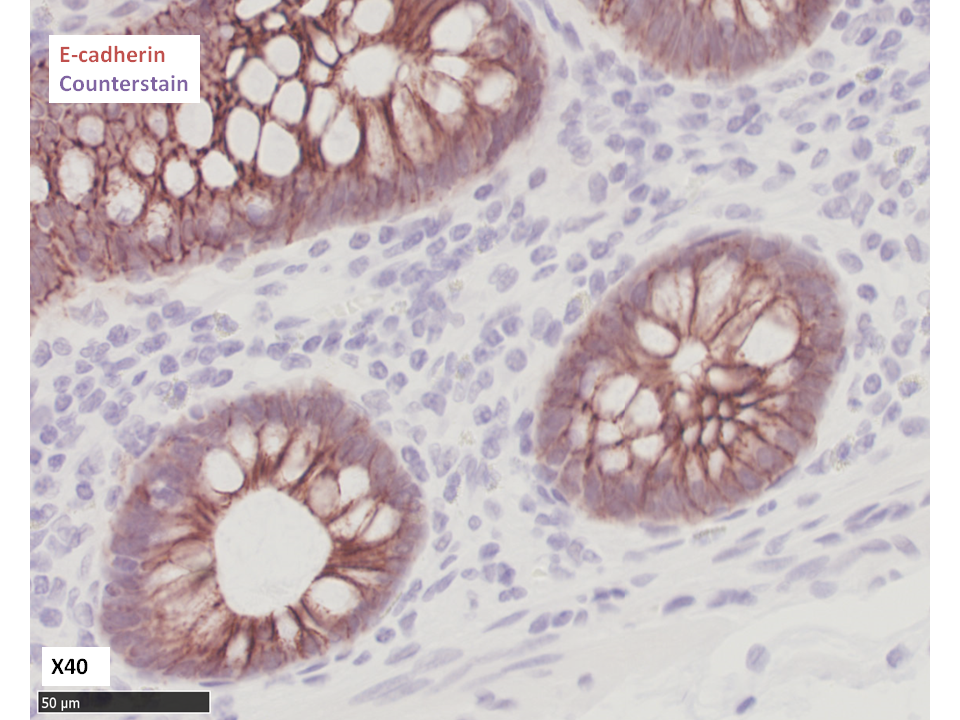
*

**Supplementary Fig. S1. E-cadherin immunohistochemistry.** Representative images of immunohistochemical staining demonstrating specific positivity for e-cadherin within the epithelium of non-strictured (A, x9.1; B, x40) and strictured (C, x9.1; D, x40) intestinal sections from patients with fibrostenosing Crohn’s disease. Panels B and D show the areas highlighted in panels A and C respectively by the black rectangles at higher power magnifications. All panels demonstrate the lack of extra-epithelial staining of e-cadherin in representative samples of both non-strictured and strictured intestinal sections.


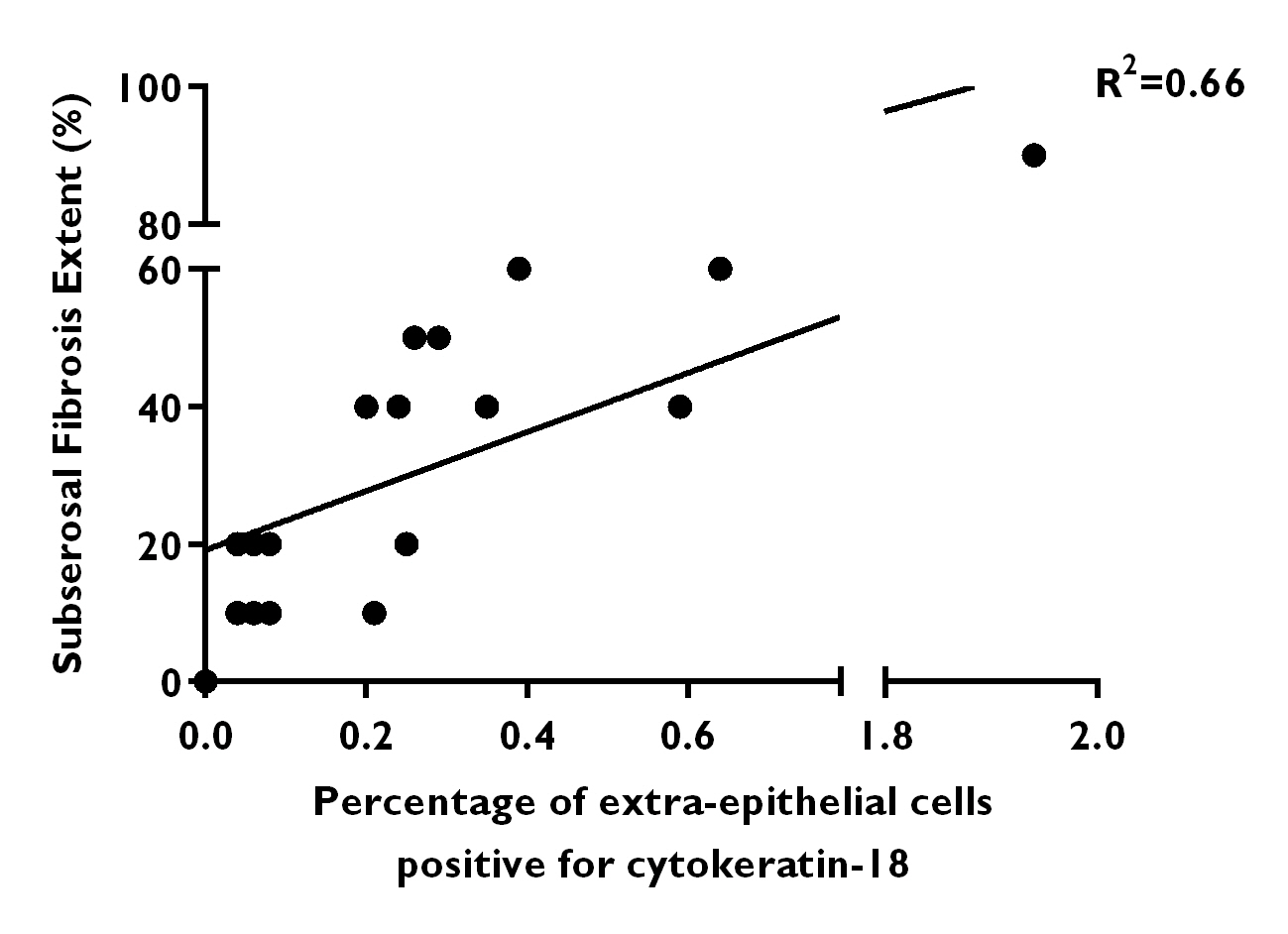


**Supplementary Fig. S2. Correlation between subserosal fibrosis extent and percentage of epithelial cells positive for cytokeratin-18.** The histological extent of subserosal fibrosis assigned by an independent expert gastrointestinal histopathologist blinded to clinicopathological details are plotted against the percentage of extra-epithelial cells positive for cytokeratin-18 staining for each sample, as analysed by the Image J programme. A positive correlation is observed (R^2^=0.66, p=0.00004). No correlations were observed between other histological scoring parameters and IHC scores.

**A**


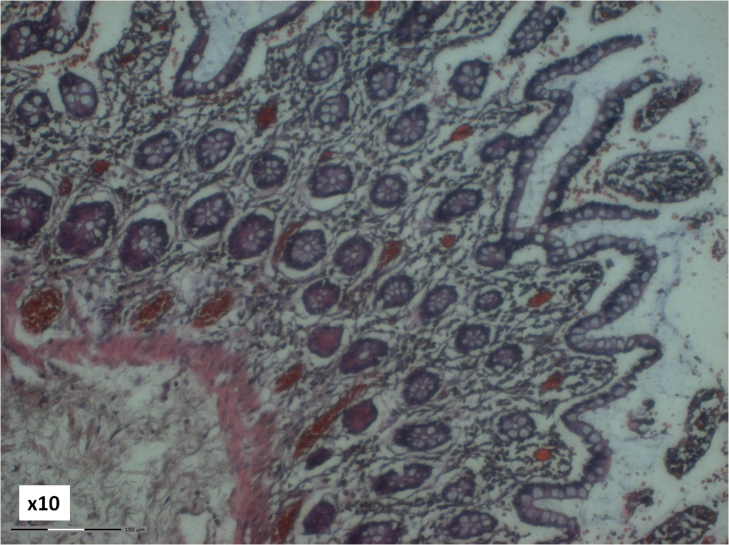


**B**

**
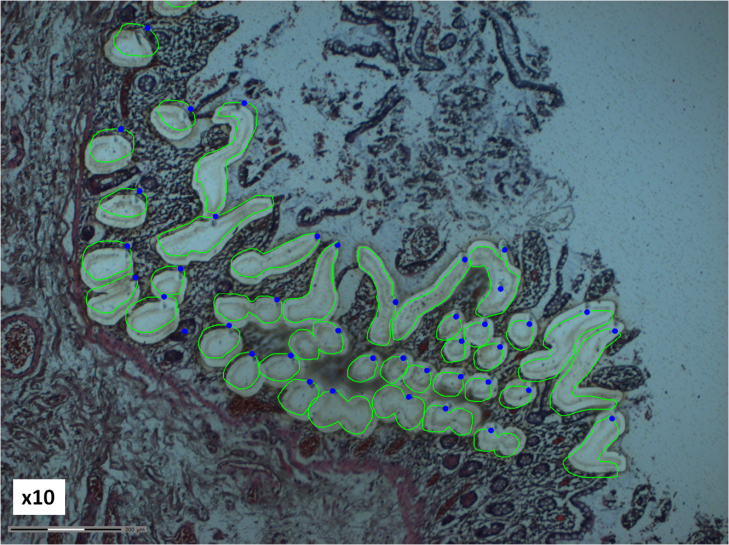
**

**Supplementary Fig. S3. Laser capture microdissection of epithelial layer from small intestinal resection specimens.**  Each FFPE section was de-paraffinised and very briefly stained with haematoxylin and eosin (Panel A). Epithelial crypts and surface epithelial layers were then isolated using the Palm Microbeam Laser Capture Microdissector (Zeiss, Germany) and catapulted into collection tubes, leaving behind submucosa and smooth muscle (Panel B). The surface area of each element was recorded and totalled to achieve the target area (4 million µm^2^) within a 1.5% error rate. Epithelial Laser capture microdissection dissection was performed to ensure as little stromal contamination as possible.





**Supplementary Fig. S4. Haemolysis ratios of serum samples from patients with CD and healthy controls, as calculated by the difference between the normalised Ct values of miR-23a and miR-451a.** A ratio >7 is considered to be at high risk of haemolysis. 10 samples showed haemolysis ratios greater than 7, and were excluded from further analysis. One sample was excluded from the healthy control group, six from the NSCD group and three from the SCD group.

**Supplementary Tables**

**Supplementary Table S1.** Pre-determined thresholds for pigment colour, size and shape identification for ImageJ software applied to all immunohistochemistry tissue samples.

| **Parameters for counterstaining** | **Value** |
| --- | --- |
| Colour threshold: Red | 0-203 pixel intensity |
| Colour threshold: Green | 115-255 pixel intensity |
| Colour threshold: Blue | 88-255 pixel intensity |
|  |  |
| **Parameters for positive staining** |  |
| Colour threshold: Red | 1-191 pixel intensity |
| Colour threshold: Green | 0-179 pixel intensity |
| Colour threshold: Blue | 0-184 pixel intensity |
| Size | 50-50,000 pixels |
| Shape: Circularity | 0.00 – 1.00 |

**Supplementary Table S2. Non-epithelial cytokeratin-18 staining profiles in NSCD and SCD surgical resection specimens.**

| **Sample** | **NSCD** | | | | | | | | | | **SCD** | | | | | | | | | |
| --- | --- | --- | --- | --- | --- | --- | --- | --- | --- | --- | --- | --- | --- | --- | --- | --- | --- | --- | --- | --- |
|  | HPF1 | | | HPF2 | | | HPF3 | | | **Mean**  **%** | HPF1 | | | HPF2 | | | HPF3 | | | **Mean %** |
|  | T | P | % | T | P | % | T | P | % |  | T | P | % | T | P | % | T | P | % |  |
| 1 | 601 | 2 | 0.33 | 105 | 1 | 0.95 | 1047 | 5 | 0.48 | **0.59** | 477 | 19 | 3.98 | 719 | 5 | 0.69 | 88 | 1 | 1.14 | **1.94** |
| 2 | 530 | 0 | 0.00 | 936 | 7 | 0.75 | 184 | 0 | 0.00 | **0.25** | 844 | 6 | 0.71 | 561 | 2 | 0.35 | 304 | 0 | 0.00 | **0.35** |
| 3 | 583 | 0 | 0.00 | 525 | 0 | 0.00 | 124 | 0 | 0.00 | **0.00** | 732 | 14 | 1.91 | 560 | 0 | 0.00 | 306 | 0 | 0.00 | **0.64** |
| 4 | 1140 | 2 | 0.18 | 1052 | 0 | 0.00 | 1264 | 1 | 0.08 | **0.08** | 1451 | 12 | 0.83 | 848 | 0 | 0.00 | 1180 | 4 | 0.34 | **0.39** |
| 5 | 822 | 2 | 0.24 | 262 | 0 | 0.00 | 771 | 3 | 0.39 | **0.21** | 1077 | 1 | 0.09 | 1416 | 6 | 0.42 | 509 | 1 | 0.20 | **0.24** |
| 6 | 930 | 1 | 0.11 | 435 | 0 | 0.00 | 824 | 0 | 0.00 | **0.04** | 933 | 2 | 0.20 | 1091 | 0 | 0.00 | 780 | 3 | 0.38 | **0.20** |
| 7 | 763 | 1 | 0.13 | 240 | 0 | 0.00 | 682 | 0 | 0.00 | **0.04** | 833 | 0 | 0.00 | 886 | 2 | 0.23 | 200 | 0 | 0.00 | **0.08** |
| 8 | 480 | 0 | 0.00 | 546 | 1 | 0.18 | 786 | 0 | 0.00 | **0.06** | 498 | 1 | 0.20 | 317 | 1 | 0.31 | 369 | 1 | 0.27 | **0.26** |
| 9 | 836 | 0 | 0.00 | 967 | 0 | 0.00 | 1148 | 2 | 0.17 | **0.06** | 476 | 1 | 0.21 | 268 | 1 | 0.35 | 323 | 1 | 0.31 | **0.29** |
| **Mean%** | **0.15** | | | | | | | | | | **0.49** | | | | | | | | | |
| **T Test** | **0.043** | | | | | | | | | | | | | | | | | | | |

The ImageJ programme was used to calculate the total number of cells (T) and the number of positively stained cells (P) for cytokeratin-18 in three randomly selected high power fields at x20 magnification. Positively stained cells were expressed as a percentage of total cells and mean values calculated for each section. *HPF, high power field*

**Supplementary Table S3.** **Epithelial vimentin staining profiles in NSCD and SCD surgical resection specimens.**

| **Sample** | **NSCD** | | | | | | | **SCD** | | | | | | |
| --- | --- | --- | --- | --- | --- | --- | --- | --- | --- | --- | --- | --- | --- | --- |
|  | Surface Epithelium | | | Crypts | | | **Mean %** | Surface Epithelium | | | Crypts | | | **Mean %** |
|  | Total Area (pixels) | Positively Stained Area  (pixels) | % | Total Area  (pixels) | Positively Stained Area  (pixels) | % |  | Total Area  (pixels) | Positively Stained Area  (pixels) | % | Total Area  (pixels) | Positively Stained Area  (pixels) | % |  |
| 1 | 35189 | 0 | 0 | 13151 | 0 | 0 | **0** | 41897 | 2082 | 4.97 | 14016 | 853 | 6.09 | **5.53** |
| 2 | 33159 | 1886 | 5.69 | 10958 | 956 | 8.73 | **7.21** | 39658 | 2704 | 6.82 | 9523 | 1348 | 14.16 | **10.49** |
| 3 | 42310 | 4662 | 11.02 | 13006 | 1862 | 14.32 | **12.67** | 44730 | 11679 | 26.11 | 9836 | 2774 | 28.21 | **27.16** |
| 4 | 32006 | 918 | 2.87 | 16023 | 876 | 5.47 | **4.17** | 39050 | 1280 | 3.28 | 13951 | 1216 | 8.72 | **6.00** |
| 5 | 35006 | 1403 | 4.01 | 13569 | 696 | 5.13 | **4.57** | 39649 | 3810 | 9.61 | 14785 | 2358 | 15.95 | **12.78** |
| 6 | 42360 | 2888 | 6.82 | 11286 | 1338 | 11.86 | **9.34** | 36541 | 9405 | 25.74 | 14589 | 4271 | 29.28 | **27.51** |
| 7 | 37437 | 372 | 0.97 | 12068 | 549 | 4.55 | **2.76** | 40285 | 10832 | 26.89 | 10753 | 3048 | 28.35 | **27.62** |
| 8 | 34590 | 2718 | 7.86 | 9865 | 1221 | 12.38 | **10.12** | 44520 | 9847 | 22.12 | 13520 | 3723 | 27.54 | **24.83** |
| 9 | 38510 | 2106 | 5.47 | 10621 | 1326 | 12.49 | **8.98** | 40453 | 3738 | 9.24 | 14531 | 2894 | 19.92 | **14.58** |
| **Mean %** | **6.65** | | | | | | | **17.39** | | | | | | |
| **T test** | **p = 0.003** | | | | | | | | | | | | | |

The ImageJ programme was used to calculate the total area assessed and the area positively stained for vimentin in three randomly selected crypts and two randomly selected regions of one hundred consecutive surface epithelial cells at x20 magnification. Mean area figures for crypts and surface epithelium for each section are shown here. Positively stained areas were expressed as a percentage of total area and mean values calculated for each section. The latter was expressed as a percentage of the former. The mean percentages are shown for each group (NSCD v SCD) with the p value as calculated using a Student’s t test showing statistical significance.

**Supplementary Table S4. Histological scoring system.**

Each sample was graded by an independent gastrointestinal histopathologist blinded to clinical details, according to the severity and extent of submucosal and subserosal fibrosis, with scores for both groups summated for a total fibrosis score for each sample. Ulcers, if present, were scored in the same way. *NA, not applicable; MP, muscularis propria; MM muscularis mucosa.*

**Supplementary Table S5. Tissue compartment areas isolated for each sample for laser capture microdissection.**

| **Sample** | **Epithelial area (µm^2^)** | **Submucosal area (µm^2^)** | **Smooth muscle area (µm^2^)** |
| --- | --- | --- | --- |
| Patient 1, NSCD, A | 4,002,087 | 5,845,530 | 10,101,289 |
| Patient1, NSCD, B | 3,961,527 | 6,199,108 | 10,117,933 |
| Patient 1, NSCD total | 7,963,614 | 12,044,638 | 20,219,222 |
| Patient 1, SCD, A | 3,999,462 | 5,892,231 | 9,905,634 |
| Patient 1, SCD, B | 4,004,826 | 6,060,763 | 10,167,364 |
| Patient 1, SCD total | 8,004,288 | 11,952,994 | 20,072,998 |
| Patient 2, NSCD, A | 4,001,397 | 6,009,124 | 9,879,851 |
| Patient 2, NSCD, B | 4,000,512 | 5,980,931 | 10,535,109 |
| Patient 2, NSCD total | 8,001,909 | 11,990,055 | 20,414,960 |
| Patient 2, SCD, A | 3,921,047 | 6,167,047 | 10,484,810 |
| Patient 2, SCD, B | 3,928,829 | 5,998,099 | 10,310,017 |
| Patient 2, SCD total | 7,849,876 | 12,165,146 | 20,794,827 |
| Patient 3, NSCD, A | 4,002,957 | 6,124,699 | 9,898,044 |
| Patient 3, NSCD, B | 3,904,611 | 6,246,450 | 10,481,020 |
| Patient 3, NSCD total | 7,907,568 | 12,371,149 | 20,379,064 |
| Patient 3, SCD, A | 4,000,919 | 5,956,216 | 10,027,145 |
| Patient 3, SCD, B | 4,007,747 | 5,947,185 | 9,970,307 |
| Patient 3, SCD total | 8,008,666 | 11,903,401 | 19,997,452 |

A block of strictured small intestinal tissue and a second block of normal small intestinal tissue were identified from three separate patients with Crohn’s disease. Two sections were cut from each block (‘A’ and ‘B’). Laser capture microdissection was used to isolate the epithelial, submucosal and smooth muscle compartments from each section. The isolated areas for sections ‘A’ and ‘B’ for each block were then combined for subsequent RNA extraction. The target for epithelial compartment area was set at 4 million µm^2^ as this was the available area from the section with the shortest segment of epithelium. The combined area was therefore 8 million µm^2^. The same principal was applied to the submucosa, where the area of the small section available for harvest was 6 million µm^2^, giving a combined area of 12 million µm^2^. Finally, the target for the muscle compartment was 10 million µm^2^ per section, giving a combined area of 20 million µm^2^. Larger areas of submucosa and muscle were harvested to allow greater RNA yield form these compartments. Areas were standardised to within a 1.5% error between samples.

**Supplementary Table S6. Total tissue compartmental areas isolated by laser capture microdissection for strictured and non-strictured groups.**

| **Tissue**  **section** | **Epithelial**  **area (µm^2^)** | **Submucosal**  **area (µm^2^)** | **Smooth muscle area (µm^2^)** |
| --- | --- | --- | --- |
| **NSCD** | 23,873,091 | 36,405,842 | 61,013,246 |
| **SCD** | 23,862,830 | 36,021,541 | 60,865,277 |
| **Percentage difference**  **(from NSCD area)** | -0.04% | -1.05% | -0.24% |

The target areas per section for harvesting by laser capture microdissection were 4 million µm^2^, 6 million µm^2^ and 10 million µm^2^ for epithelium, submucosa and smooth muscle respectively, to within a 1.5% error. The total epithelial area isolated from the section with the smallest area available was 4 million µm^2^ and so this was used as the target area for the epithelial compartment. It was possible to harvest larger areas of submucosa and muscle and so larger target areas were chosen. Comparisons were to be made within, rather than across, compartments. Total areas harvested for each compartment for non-strictured and strictured samples are shown. Percentage differences of total area in strictured samples compared to non-strictured samples are also shown.

**Supplementary Table S7. Correlations between miR-200 family expression in different tissue compartments and histological fibrosis grades.**

| **Tissue**  **Compartment** | **miRNA** | **Histological fibrosis grade parameter** | | | | |
| --- | --- | --- | --- | --- | --- | --- |
|  |  | **Submucosal**  **extent** | **Submucosal**  **score** | **Subserosal**  **extent** | **Subserosal**  **score** | **Total fibrosis**  **score** |
| **Epithelium** | **miR-141** | 0.08 | 0.21 | 0.06 | **0.02** | 0.09 |
|  | **miR-200a** | 0.09 | 0.97 | 0.23 | 0.06 | 0.41 |
|  | **miR-200b** | 0.07 | 0.12 | 0.07 | 0.09 | 0.06 |
|  | **miR-200c** | 0.06 | 0.12 | 0.53 | 0.32 | 0.13 |
|  | **miR-429** | 0.14 | 0.32 | 0.87 | 0.23 | 0.13 |
| **Submucosa** | **miR-141** | 0.27 | 0.23 | 0.23 | 0.09 | 0.14 |
|  | **miR-200a** | 0.56 | 0.05 | 0.75 | 0.27 | 0.09 |
|  | **miR-200b** | 0.21 | 0.015 | 0.013 | 0.42 | 0.28 |
|  | **miR-200c** | 0.20 | 0.11 | 0.24 | 0.53 | 0.97 |
|  | **miR-429** | 0.25 | 0.51 | 0.23 | 0.39 | 0.71 |
| **Smooth muscle** | **miR-141** | 0.15 | 0.23 | 0.24 | 0.13 | 0.14 |
|  | **miR-200a** | 0.30 | 0.40 | 0.75 | 0.27 | 0.15 |
|  | **miR-200b** | 0.87 | 0.51 | 0.74 | 0.09 | 0.08 |
|  | **miR-200c** | 0.43 | 0.11 | 0.24 | 0.50 | 0.14 |
|  | **miR-429** | 0.20 | 0.45 | 0.23 | 0.06 | 0.35 |

Expression of members of the miR-200 family was measured in different tissue compartments of both strictured and non-strictured intestinal tissue using RT-qPCR. Pearson’s correlation coefficients between compartmental miR-200 expression (expressed as cycle threshold values) and composite histological scores of fibrosis were calculated and significance tested. P values of each correlation are shown in the table below. A significant correlation was observed between epithelial miR-141 expression and subserosal fibrosis score (in bold); no other significant correlation was observed.
